# Consumption of the Food Groups with the Revised Benefits in the New WIC Food Package: A Scoping Review

**DOI:** 10.3390/nu17050856

**Published:** 2025-02-28

**Authors:** Qi Zhang, Priyanka T. Patel, Bidusha Neupane, Caitlin M. Lowery, Futun Alkhalifah, Faezeh Mahdavi, Esther May Sarino

**Affiliations:** 1 Joint School of Public Health, Macon & Joan Brock Virginia Health Sciences at Old Dominion University, Norfolk, VA 23529, USA; ppate008@odu.edu (P.T.P.); bneup001@odu.edu (B.N.); falkh003@odu.edu (F.A.); fmahd001@odu.edu (F.M.); 2 Gillings School of Global Public Health, University of North Carolina at Chapel Hill, Chapel Hill, NC 27599, USA; clowery@unc.edu; 3 Brickell Medical Sciences Library, Macon & Joan Brock Virginia Health Sciences at Old Dominion University, Norfolk, VA 23529, USA; sarinoem@odu.edu

**Keywords:** WIC, maternal and child health, infant foods, milk, juice

## Abstract

Background: On 18 April 2024, the United States Department of Agriculture (USDA) published the first food package changes to the Special Supplemental Nutrition Program for Women, Infants, and Children (WIC) in over a decade, which reduced some food benefits (juice, milk, canned fish, and infant fruits and vegetables) and offered substitutes (cash-value vouchers (CVVs) or cash-value benefits (CVBs) to redeem for fruits and vegetables, cheese, soymilk, or other dairy products). Methods: To assess the impact of the changes on the consumption and redemption of these food groups, a systematic search was conducted, identifying 23 peer-reviewed publications between 2010 and 2024. Results: They revealed significant shifts in consumption after the 2009 food package changes; e.g., a decline in 100% juice intake following reductions in juice allowances. Additionally, the review highlighted that the 2009 WIC food package revision was associated with more fruit and vegetable consumption after the increase in CVV allowance. While including milk alternatives like soy-based beverages or lactose-free milk or cheese may improve redemption rates and WIC program satisfaction, the long-term impacts of these proposed changes remain unknown. No research was identified on the consumption of canned fish. Conclusions: This review contributes to understanding the changes in redemption and consumption after the last WIC food package changes, identifies the knowledge gap about prospective impacts, and recommends that the WIC agencies implement appropriate evaluations to promote health and nutrition among vulnerable populations.

## 1. Introduction

In November 2022, the U.S. Department of Agriculture (USDA) proposed once-a-decade major rule changes regarding the food benefits of the Special Supplemental Nutrition Program for Women, Infants, and Children (WIC) [1]. After extensive public comment, the USDA published the final rules on 18 April 2024 [2]. These rule changes were based on recommendations from the expert panel of the National Academies of Sciences, Engineering, and Medicine (NASEM) [3]. As a way to improve the nutritional quality of the WIC food package, several food groups’ benefits, including juice, milk, canned fish, and infant fruits and vegetables, were reduced or eliminated and replaced with alternative food benefits, such as lactose-free milk, yogurt, soy-based beverages, cheese, and a cash-value benefit (CVB) or cash-value voucher (CVV) to redeem for fruits and vegetables [1,3] (see Table 1 for details on changes in the final rules and their associated rationales). Reducing WIC benefits for specific food groups may lead to concerns about its potential impact on the nutritional intake of participants. While increasing CVB for fruits and vegetables is a positive step, reducing other food benefits could result in deficiencies in essential nutrients, particularly for vulnerable populations. Providing substitutes to these food groups may help address the concern. However, it is not clear how these specific food group changes would affect the participants’ redemption and consumption, which requires further research on the consumption of these food groups among WIC participants.

Understanding these food groups’ changes in redemption and consumption can help the USDA develop relevant programs and interventions to ensure that the program can achieve its goal of promoting the health of vulnerable women, infants, and children in the U.S. However, a formal assessment of these impacts requires a long waiting period after full implementation, making it challenging to adopt a prospective design of the program evaluation. Moreover, rigorous evaluations, including a potential simulation of these reduction and substitution effects, require a thorough understanding of previous research. Researchers can adequately design and implement future assessments if a comprehensive review identifies knowledge gaps. While previous systematic reviews have been conducted to examine the impact of the 2009 WIC-revised food package on dietary intake, breastfeeding outcomes, and fruit and vegetable consumption and redemption [4,5,6], no comprehensive review has focused on all food categories affected by the policy change. Given the time sensitivity and knowledge gap, this scoping review summarizes evidence on the consumption and redemption of food groups, including milk, juice, canned fish, and dairy substitutes, to provide a broader perspective on food benefits after the 2009 WIC revision.

## 2. Materials and Methods

Due to the exploratory nature of the review on a broad topic, we conducted a scoping review between September and October 2024, utilizing four databases: PubMed, Ovid Medline, APA PsycInfo, and CINAHL (Cumulative Index of Nursing and Allied Health Literature). The research team, including a medical librarian, developed a comprehensive search strategy using various combinations of medical subject headings (MeSH) and key terms, such as “WIC”, “diet”, “consumption”, “nutrition”, “women”, and “child”, to focus on the research topic. The librarian constructed a search strategy based on the agreed-upon terms and searched the above databases on 8 October 2024. Table 2 provides the general mapping of the Medical Subject Headings (MeSH) and keywords (see Appendix A for the search strategies). The inclusion criteria were articles published in English in peer-reviewed journals between 2010 and 2024. The year 2010 was purposefully chosen as the year following the 2009 WIC package revision. Studies conducted outside the United States were excluded. This review focused on WIC participants and their consumption and/or benefit redemption of the selected food categories (including milk, cheese, and other WIC-eligible dairy products; juice; infant fruits and vegetables; canned fish; and fruits and vegetables). Studies were included if they compared WIC participants with other non-WIC peers (difference-in-difference design), compared before and after the 2009 food package revision, or provided cross-sectional insight into consumption and redemption patterns. Studies on the CVB/CVV value increase to purchase fruits and vegetables during the COVID-19 pandemic were excluded.

**Figure 1:**
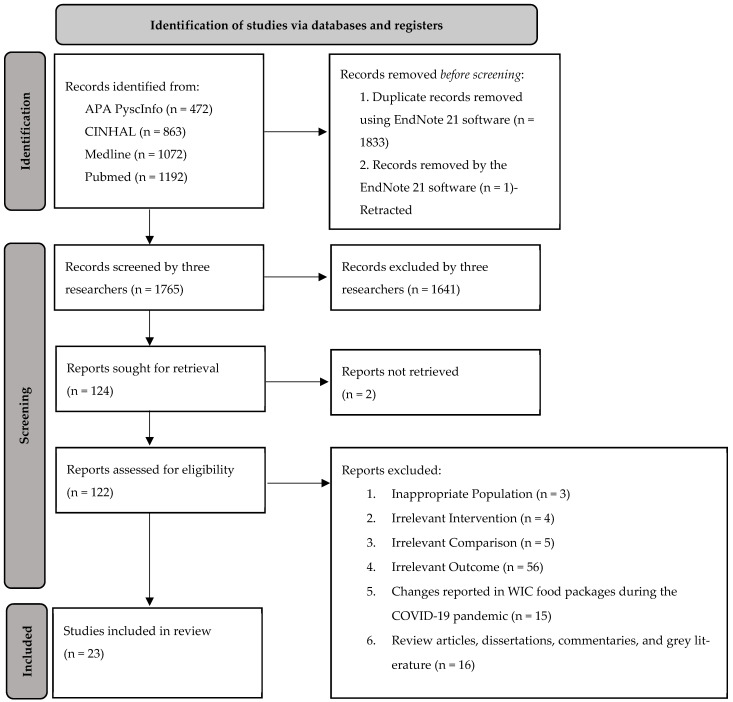
Flow diagram of studies included in the scoping review. Notes. Adapted from Page MJ, McKenzie JE, Bossuyt PM, Boutron I, Hoffmann TC, Mulrow CD, et al.: “PRISMA 2020 flow diagram for new systematic reviews which included searches of databases, registers, and other sources”. The PRISMA 2020 statement: an updated guideline for reporting systematic reviews. BMJ, 372. doi:10.1136/bmj.n71 [7].

After retrieving the search results, all records were imported into EndNote version 21, a software for reference management and further screening. Before the title and abstract screening process, duplicate records were automatically identified and removed in EndNote 21. Any remaining duplicate articles missed by the software were manually removed during the title and abstract screening process. Three researchers independently screened 1765 titles and abstracts to assess their eligibility and relevance to the review, following the PRISMA guidelines [7]. A total of 124 articles were identified for full-text review; however, two articles were excluded due to their inaccessibility. Upon thoroughly reviewing 122 full texts, 99 articles were not included in the study based on the inclusion criteria. Ultimately, 23 publications were included in this scoping review, and their quality was assessed based on the Academy of Nutrition and Dietetics guidelines in systematic reviews [8]. Each study was evaluated based on the appropriateness of the research design and the overall quality of the implementation of the research design. The quality criteria checklist covered 10 domains for validating the quality of a study: research questions, subject selection, comparable groups, withdrawals, blinding (if applicable), intervention/exposure, outcomes, analysis, conclusion support, and the likelihood of bias. Based on these criteria, each study was rated as positive, neutral, or negative [8]. Two researchers independently assessed the quality of the papers, and any discrepancies were resolved by discussing them with the entire research team. The selection process is illustrated in Figure 1. Data from each included publication were extracted into a structured form using the PRISMA table guideline, which includes author(s), publication year, locations, food category, primary outcomes, key findings, and quality assessment. Discrepancies between researchers at each stage of the review process were resolved through discussion with the research team.

## 3. Results

Table 3 summarizes the study characteristics and results of all 23 articles in this review [9,10,11,12,13,14,15,16,17,18,19,20,21,22,23,24,25,26,27,28,29,30,31]. The quality of 22 studies was positive, with only one study with neutral quality [14]. The years of the included articles ranged from 2012 to 2023, with peak publication years in 2014 [13,14,15,16] and 2015 [17,18,19,20], each with four articles. Six articles included a national sample [24,25,26,28,29,31]. Among the other regional studies, New England states had five articles (the highest number of studies) [10,11,13,17,21]. Two studies were conducted each in California [9,14], Illinois [15,16], Texas [20,22], and Virginia [23,30]. Regarding the food categories, both milk [9,12,13,15,16,18,19,21,22,24,25,27,28,29,31] and juice [10,11,14,15,16,19,20,21,22,24,25,26,27,28,29] were the most studied among the food categories, with 15 articles. Fruits and vegetables had 14 articles [9,12,15,16,17,19,20,24,25,26,28,29,30,31]. Four articles included infant fruits and vegetables [20,23,24,29]. No articles targeted the consumption or redemption of canned fish as the study outcome. Given that canned fish can provide essential omega-3 fatty acids and protein [32], the lack of research on this food group is a notable gap, which may overlook important nutritional aspects and result in a potential compromise of the participants’ dietary intake. This review revealed a high interest in consumption, with 15 articles studying consumption [9,12,14,15,16,18,19,20,22,24,26,27,28,29,31], and eight articles studying redemption [10,11,13,17,21,23,25,30].

Regarding the study designs, 12 studies used an observational design [10,12,20,22,23,24,26,27,28,29,30,31] and one study used the qualitative approach [14], which limits the interpretation of the causal relationship between the 2009 WIC food package revision and the outcomes. Ten studies adopted the pre–post design with various statistical analyses to evaluate the association between the food package change and participant outcomes, including inference tests [9,16], mixed effects methods [19], Generalized Estimating Equations (GEE) [11,13,15,17,21], and difference-in-difference models [25]. One study adopted a longitudinal design with three surveys before and after the 2009 food package revisions over four weeks, which was essentially a pre–post study [18]. Therefore, no study adopted a more rigorous experimental design or statistical techniques, such as instrumental variables, regression discontinuity, or inverse probability weighting, to estimate the causal effects of the 2009 WIC food package revisions on the consumption or redemption of the selected food groups.

## 4. Discussion

This scoping review summarizes findings from studies with different study designs to obtain deeper insight into the topic, including pre–post comparisons, WIC and non-WIC participant comparisons, and cross-sectional analyses. While causal inference cannot be drawn, all included studies provide a comprehensive understanding of the food redemption and consumption patterns following the 2009 food package revision, which is consistent with the prior systematic reviews [4,5,6].

### 4.1. Fruits and Vegetables

Overall, the CVV addition to the WIC food package was positively associated with consuming and redeeming fruits and vegetables among WIC participants [12,17,29]. For instance, Guthrie et al. [29] found increased fruit and vegetable consumption among WIC children between 2008 and 2016, indicating a positive change in dietary behaviors. However, a few studies indicated no significant change or even a decrease in vegetable consumption [15,19]. For example, Morshed et al. [19] specifically noted a decrease in vegetable consumption (excluding potatoes) in rural New Mexico, while fruit consumption remained unchanged. Vercammen et al. [26] reported no significant difference in the consumption of whole fruits and vegetables between WIC participants and non-participants.

The revision of the WIC food package also led to increased purchases of fruits and vegetables and higher program satisfaction, perceived program value, and preference for CVVs for fruits and vegetables [33]. According to Andreyeva and Luedicke [17], WIC households in New England states spent three times more on fresh fruit than fresh vegetables. Similarly, two studies observed small but significant increases in purchasing and consuming fruits and vegetables [9,25]. In alignment with the purchase outcome, fresh produce accounted for most CVB redemptions. A study by Zhang et al. [30] highlighted and documented a 77.3% redemption rate of fresh produce in Virginia. These results suggest that adding CVVs into the food package consistently increased the purchase of fruits and vegetables across different regions and populations.

### 4.2. Infant Fruits and Vegetables

The 2009 WIC food package revision added infant fruits and vegetables, i.e., purees, into the infant packages to increase their exposure to fruits and vegetables [2]. WIC infants’ overall fruit consumption was not statistically different compared to lower-income, non-participating infants. Still, they consumed more infant fruits, i.e., less fresh fruit, than their non-participating counterparts [24]. The infant vegetable benefit resulted in a higher prevalence of vegetable consumption among WIC infants. For example, among infants aged 6 to 11.9 months, 74% consumed any vegetable compared with 61% of low-income, non-participating infants. The gap between infant vegetables was more significant (55% vs. 32% between WIC and non-WIC infants, respectively) [24]. In another smaller-scale study, researchers did not find significant changes in exposure to infant fruits and vegetables among WIC infants between 2009 and 2011 [20], partially due to the short observation period after the 2009 WIC food package change. However, a national study found a higher prevalence of WIC infants aged 6 to 11.9 months in 2016 than in 2008 (56% vs. 41% respectively) who consumed infant fruits, compared with a lower prevalence of non-WIC infants who consumed infant fruits during the same period (39% vs. 52%, respectively) [29]. A higher percentage of WIC infants consumed infant vegetables during that period (55% in 2016 vs. 29% in 2008), while a lower percentage of non-WIC infants consumed the same food at the same time (30% in 2016 vs. 47% in 2008) [29].

The full redemption of infant fruits and vegetables has remained low. For instance, 37.5% of Michigan WIC households redeemed all infant fruits and vegetables in 2014 [34]. Although the benefits are free, research suggests that participants prefer higher-priced brands to lower-priced ones [23]. Although most mothers were satisfied with infant fruits and vegetables obtained at no cost, many still prefer fresh fruits and vegetables, which can be purchased with the cash-value benefit (CVB) [35]. Therefore, based on some of the study findings, substituting infant fruits and vegetables with a CVB may increase fruit and vegetable benefits redemption; however, further research is needed to improve participant program satisfaction.

### 4.3. Juice

The included studies collectively suggested a reduction in the consumption of 100% juice among WIC children after the juice allowance was reduced in the 2009 WIC food package revision, although the magnitude of the reduction varied across outcomes and populations [14,15,16,19,20,22,25]. For instance, the study by Beck et al. [14] in 2014 from California highlighted barriers to juice consumption within the WIC population, citing a reduction in juice benefit allowance by WIC as a potential cause of juice consumption reduction. Similarly, studies by Odoms-Young et al. [16], Morshed et al. [19], and Reat et al. [20] found no significant change in 100% fruit juice consumption. Kong et al. [15] reported declining juice consumption in Chicago among Hispanic mothers. However, several studies documented that WIC children consumed more 100% juice compared to non-WIC participants [22,24,26,27,28,29]. Charvet et al. [27] reported that WIC children in Florida consumed more than twice the recommended amount of 100% fruit juice daily. Encouragingly, Hamner et al. [28] reported that while WIC children consumed more juice than recommended, the overall trends in consumption were downward. Households with WIC-only children purchased a larger share of the fruit beverage volume of 100% juice than those with both WIC and the Supplemental Nutrition Assistance Program (SNAP) benefits [10].

In contrast to the consumption trend of 100% juice, WIC participation was associated with lower juice intake during the decline in consumption of high sugar-sweetened beverages in general populations. Andreyeva et al. [11] documented a significant decrease in 100% juice purchases by WIC households, which aligned with the program’s objective of reducing juice consumption. The findings of Andreyeva et al. [21] and Ng et al. [24] aligned with this trend toward lower juice purchasing by reporting decreases in the purchases of sugar-sweetened drinks. The decline in juice consumption can vary across ethnic groups. For example, Hispanic mothers had a significant decrease in juice consumption [15,16] but Hispanic or African American children or African American mothers did not have a significant reduction six months after the implementation [16]. The decline of 100% juice consumption among WIC participants may not be fully attributable to the 2009 WIC food package revision. In an evaluation of household purchases of WIC-participating and income-eligible non-participating households, both groups reduced their purchases of 100% juice during this period, indicating that juice consumption may be part of dietary preference rather than merely a result of food package revision [25]. Despite these positive changes, a qualitative study conducted by Beck et al. [14] in California revealed a communication gap. The author stated that WIC caregivers expressed confusion about why WIC provides juice when they advise parents to avoid giving their children juice to prevent them from dental caries, diabetes, and obesity.

One hundred percent juice is an important source of fruit for young children. The 2009 WIC food package revision provided 128 fl. oz./3.78 L of 100% juice monthly for children aged 1 to 4 y [36]. Since 8 fl. oz./0.23 L or one cup of 100% juice is approximately one cup of whole fruit [37], 100% juice benefits allow for approximately 0.5 cups of whole fruit daily, meeting 33% to 50% of the recommended fruit intake among young children based on the 2010 Dietary Guidelines for Americans [38]. The 2024 WIC food package revision reduced the 100% juice benefits for children to 64 fl. oz./1.89 L but increased the CVB from $9 to $26/month to purchase fruits and vegetables [39]. Since the volume of the fruits and vegetables to be redeemed with CVB depends on the unit price, the impact of this reduction and substitution between 100% juice and CVB on WIC children’s fruit consumption deserves more careful examination. A recent simulation study suggested that replacing juice with whole fruit among children 1–5 y would reduce total energy and sugar intake but increase fiber and have little impact on vitamin C and calcium. However, the replacement strategy would increase daily food costs by $0.44–0.49 [40]. Therefore, the increase in CVB for WIC children might be able to cover higher whole fruit costs adequately, although the increasing cost of fresh fruits threatens the coverage [41].

### 4.4. Milk and Dairy Products

The 2009 WIC food package revision reduced milk allowance by 1 gal/3.79 L, 1.5 gal/5.68 L, 2 gal/7.57 L, and 3 gal/11.36 L for WIC mothers depending on their specific participant categories (i.e., exclusive breastfeeding, pregnant, partial breastfeeding/postpartum, respectively) and children [15,42]. The milk benefit reduction presents an interesting dilemma between preferences, redemption, and consumption of whole and reduced-fat milk among participants. Following the WIC 2009 revisions, there has been a notable shift from whole milk to low-fat milk among WIC participants, particularly among children [18] and certain ethnic groups, such as Hispanics and African Americans [13,15,16,21]. For example, the prevalence of Texas WIC children aged 2–4 y consuming whole milk decreased from 61.4% to 8.7%, while their consumption of reduced-fat milk increased from 32.6% to 81.5% [22]. Furthermore, Guthrie et al. [24] and Charvet et al. [27] underscored the challenge of milk overconsumption, with WIC children consuming more than the recommended amounts of low-fat milk, pointing to potential gaps in education about portion sizes. In addition, Guthrie et al. [24] reported that children of different age groups have shown distinct consumption patterns, with younger children drinking more whole milk and older ones drinking more low-fat and non-fat milk.

In response to the WIC food package revisions, there has been a significant shift in milk redemption patterns among participants, notably moving toward lower-fat milk choices. The purchase volume of higher-fat milk decreased during the post-revision period for both WIC and non-WIC households; however, the decrease in WIC households was significantly larger than in non-WIC households (37% vs. 13%, respectively) [25]. Similarly, Andreyeva et al. [13] observed a substantial decrease in the redemption of high-fat milk, which aligns with the revisions’ aim to promote the consumption of lower-fat milk options. However, the redemption rates of whole milk were greater than those of low-fat milk in West Virginia and Kansas. For example, the redemption rates for whole milk were 69.3% in Kansas and 75.4% in West Virginia, while the rates for low-fat milk were 57.6% and 59.1%, respectively [43,44].

The reduction of milk allowance in the WIC food package revision has been associated with notable changes in milk consumption. However, it appears to be less associated with participants’ changing milk preferences. WIC participants overwhelmingly preferred whole milk over reduced-fat milk, even after the revision [33]. According to Whaley et al. [9], in California, these policy adjustments were associated with a decrease in whole milk intake and an increased preference for low-fat milk among WIC participants, suggesting an alignment of consumption with the desired health objectives of the program. However, Kong et al. [15] in Chicago documented that while the milk consumption of African American and Hispanic children shifted toward reduced-fat options, there was no significant decrease in whole milk consumption among all groups. Therefore, post-2009 food revision evidence suggests that a reduction in milk allowances was associated with lower whole milk consumption among participants. However, studies did not necessarily report changes in the participants’ preferences or redemption patterns.

Regarding other dairy products, an overwhelming majority of WIC children’s caregivers (93%) in a qualitative study would like or very much like cheese in the WIC food package [33]. However, a national study of WIC children in 2016 found that 36% of WIC children 12–23.9 m and 40% of those 24–47.9 m consumed WIC-eligible cheese, and the prevalence was not statistically different from low-income non-WIC peers [24]. Moreover, a higher prevalence of the same WIC children consumed cheese in 2016 (34%) than in 2008 (28%), but the trend was not significantly different from that in non-WIC peers (39% in 2016 vs. 32% in 2008) [29]. A small study in Florida (n = 197) found only 5.1% of WIC preschoolers consumed soy or other milk, i.e., non-whole or lower-fat milk [26], whereas WIC children 12 to 23 months consumed less dairy, including milk, yogurt, and cheese, than eligible non-participants [28]. Another study of 515 WIC households in Connecticut found that the purchase volume of WIC-eligible cheese decreased by 37.2% from 2009 to 2010 following the implementation of the WIC revised food package [13]. The review identified little research that focused on the consumption or redemption of yogurt as a separate food group from other dairy food groups. Therefore, the effect of substituting milk with other dairy products was uncertain and should be further studied.

This review’s results need to be interpreted in a setting with significant operational changes made to the WIC program from 2009 to 2024. For example, the WIC program used paper-based vouchers with printed food benefits on them in 2009, while the Electronic Benefit Transfer (EBT) was implemented in most WIC state agencies in 2022, except a few Indian Tribal Organizations (ITOs) [1], which facilitated the WIC households’ redemption of benefits [30,45,46]. The WIC shopping apps reflected the benefits balance and their usage was associated with higher redemption rates among these food groups among WIC households [43,44]. Moreover, nutrition education’s impact on food package changes deserves further scrutiny. For example, California WIC launched a statewide campaign on nutrition education to promote fruits and vegetables, whole grains, and low-fat milk consumption before the 2009 WIC food package changes, which was associated with a high acceptance of the education message and more consumption of these food groups [47]. However, immediately after implementing the new food packages, California WIC focused the education entirely on using the new checks instead of on food consumption, so the real impact of nutrition education on the revised food package was unknown [9]. Therefore, the 2024 WIC food package revisions will be informational in studying the pattern of consumption and redemption of the selected food group by the WIC participants. In addition, further research is needed to implement measurements of the magnitude of its impact in a timely fashion.

### 4.5. Limitations of the Research Reviewed

The studies reviewed offer valuable insights into the consumption and redemption of selected food groups after WIC food package revisions in 2009, yet they collectively are subject to a number of limitations. Geographical variability across the studies underscores a key limitation, as many studies were conducted from a limited scope, such as those focusing exclusively on participants from specific states like Texas, California, and Illinois, or demographically, with a significant number examining predominantly Hispanic populations.

Another critical issue is the reliance on self-reported data, which is prone to social desirability bias. The use of observational designs also limits the ability to determine causality. Without an experimental design, attributing observed dietary changes directly to WIC policy revisions remains challenging. The applications of some statistical techniques, such as instrumental variables, inverse probability of treatment weighting, and regression discontinuity, may remedy the problem and draw causal connections between the WIC food package revisions and consumption or redemption of the food groups.

Many studies overlook actual food consumption, relying instead on purchase data or observing short-term changes, which may not reflect sustained dietary habits. Furthermore, the research focuses on specific food categories like milk, fruits, and vegetables; while valuable, these categories only encompass part of the range of dietary intake. Therefore, the review results should be carefully interpreted due to changing baseline food packages, package revisions, and operational changes in the WIC program from 2009 to 2024, such as shifting from paper-based vouchers to EBT cards and other technology innovations, such as the WIC shopping apps.

In summary, while the reviewed studies provide valuable insights into the effectiveness of nutritional interventions and the behaviors of specific populations, the noted limitations highlight the necessity for broader, more inclusive, and methodologically rigorous research to fully understand the complexities of dietary behaviors and program impacts across diverse communities.

### 4.6. Policy Implications and Future Research

Reviewing studies on the WIC food package revisions reveals critical policy implications and sets a direction for future research. A further decline of the 100% fruit juice category is expected, but whether the total fruit intake can be made up with the increase in CVB has yet to be examined. The complexity of these mixed results emphasizes the need for a more refined understanding of how these food package changes interact. The changes may disproportionately affect certain WIC participants, especially those who rely heavily on specific food categories. Exploring how these changes affect different groups of WIC participants is essential to ensure that all WIC participants have equitable access to nutritious food options. Additionally, introducing more flexible food packages may meet diverse dietary preferences. However, the magnitude of these benefits requires more field studies. Expanding the geographical scope of these evaluation studies is necessary to assess the revised food package’s impact comprehensively. Addressing these knowledge gaps with more rigorous study designs will help to evaluate the causal relationship between food package changes and consumption. Longitudinal studies are needed to assess whether these changes will lead to sustained improvements in dietary habits or whether participants will revert to previous consumption patterns. Continuous evaluations of the program’s impact on health and nutrition are essential to ensure that changes are beneficial and to adapt strategies as needed, highlighting the importance of evidence-based policymaking in nutrition programs.

## 5. Conclusions

After the reduction of the food benefit allowance of 100% juice and milk in the 2009 WIC food revisions, studies reported an overall decline in 100% juice and whole milk consumption. However, studies on infant fruit and vegetable redemption patterns showed mixed findings. Some studies suggested stable redemption rates, while others indicated an increase in redemption due to the rise in CVV benefits. Changes in the consumption of other food groups, such as canned fish or yogurt, are still limited. Moreover, further studies are needed to determine how changes to the WIC package affect the consumption of specific food categories among different socio–demographic groups. It will also be essential to examine whether the final rules can achieve the goal of providing participants with more nutritious and highly redeemed packages after the next WIC food package revision.

## Figures and Tables

**Table 1:** The 2024 USDA final rule on WIC food packaging related to infant fruits and vegetables, juice, milk, and canned fish [2].

Food Category	Proposed Changes	Substitution	Final Revisions Based on Public Comment	Rationale
**Juice**	Reduced allowance of 100% juice (maximum allowance is 64 oz ^b/^1.89 L)	$3 CVB ^a^	Finalize as proposed	To encourage the consumption of whole forms of fruits and vegetables instead of juice; 100% juice had lower dietary fiber than whole fruits and vegetables.
**Milk**	Reduced by 2 to 4 quarts per month for children; reduced by 6 to 8 quarts per month for pregnant and breastfeeding participants	Lactose-free milk; plain or sweetened yogurt or reduced-fat yogurt; soy-based beverages, yogurt, and cheese; milk-based cheese	Finalize as proposed	1. To “promote nutrition security and equity. Ensure additional options for participants with special dietary needs and preferences across all State agencies”. 2. To be “consistent with the Child and Adult Care Food Program (CACFP) for total sugar allowance”. 3. To allow for “flexibility, variety, and choice” for participants with allergies, lactose intolerance, or a vegan diet. Soy-based beverages are considered part of the dairy group as they have equivalent nutritional content to milk. The rule includes nutrient-minimum specifications for calcium, protein, and vitamin D.
**Infant Fruits and Vegetables**	Reduced allowance (from 256 oz/7.57 L to 128/3.78 L oz per month for both fully breastfed and partially breastfed/fully formula-fed infants)	$10 (64 oz/1.89 L) or $20 CVV ^c^ (full 128 oz/3.78 L)	Finalize as proposed	To bring the amount of infant fruits and vegetables allowed to an appropriate level for daily consumption for infants (6–11 months).
**Canned Fish**	Reduced the amount of canned fish to fully breastfeeding participants from 30 oz/850 g to 20 oz/567 g	Legumes and peanut butter at 3 month-rotations	Finalize as proposed	To maintain cost neutrality.

^a^ CVB = Cash-Value Benefits. ^b^ oz = ounces. ^c^ CVV = Cash-Value Voucher.

**Table 2:** Mapping of the study Medical Subject Headings (MeSH) and keywords.

Concept	Medical Subject Headings (MeSH)	Keyword(s)
1	Food assistance	Women infants and children; WIC; special supplement * nutrition program for women, infants, and children; food program
	Food packaging	Food package *
2	Consumer behavior	Allowance *
	Food security	Food intake
	Food preferences	Food consumption/consum *
	Diet, food, and nutrition	Redeem
	Child nutritional physiological phenomena	Redemption, purchase
	Feeding behavior	Choice
	Choice behavior	Dietary practice
	Diet, food, and nutrition	Nutrition program
	Recommended dietary allowances	Eating habit *, benefit, diet *, nutrition *
3	Mothers	Child *
	Female	Baby/babies
	Child, Preschool	Women/woman/female */mother *

(*)—a truncation operator used in the database search to capture variations of the keyword used. For example, “supplement*” retrieves “supplement”, “supplements”, and “supplementation”.

**Table 3:** Summary of the study characteristics and results of the included scoping review articles on WIC ^a^ food package revisions (N = 23).

Author(s)	Year	Setting/Location	Food Category	Outcome	Result Summary	Quality *
Whaley et al. [9]	2012	California	F&V ^b^	Consumption	Fruit and vegetable consumption increased from 2009 to 2010.	Positive
			Milk	Consumption	Whole milk consumption decreased in caregivers and children, while low-fat milk consumption increased from 2009 to 2010.	
Andreyeva et al. [10]	2012	New England States	Juice	Redemption	WIC-only households purchased more 100% fruit juice beverages (by volume) than WIC households receiving SNAP ^c^ benefits.	Positive
Andreyeva et al. [11]	2013	New England States	Juice	Redemption	Total purchases of 100% juice decreased by 23.5% from 2009 to 2010.	Positive
Chiasson et al. [12]	2013	New York State	Milk	Consumption	Whole milk consumption decreased, while low-fat milk consumption increased in 2011 compared to 2008 among WIC children aged 2–4 y ^e^.	Positive
			F&V	Consumption	Daily fruit and vegetable intake increased among WIC children aged 1–4 y.	
Andreyeva et al. [13]	2014	New England States	Milk	Redemption	Whole milk redemption decreased substantially among WIC households from 2009 to 2010.	Positive
			Cheese	Redemption	Substantial decrease in WIC-eligible cheese purchases.	
Beck et al. [14]	2014	California	Juice	Consumption	WIC caregivers were confused about why WIC provides juice yet counseled parents to avoid giving children juice.	Neutral
Kong et al. [15]	2014	Chicago	Milk	Consumption	Consumption of reduced-fat milk significantly increased for African American and Hispanic children and mothers; whole milk intake significantly decreased for all groups.	Positive
			Juice	Consumption	Consumption of fruit juice was reduced among Hispanic mothers.	
			F&V	Consumption	No significant change in fruit and vegetable consumption from 2009 to 2011 in the WIC mother–child dyads.	
Odoms-Young et al. [16]	2014	Chicago	F&V	Consumption	Consumption of fruit increased among Hispanic mothers from 2009 to 2010. No changes in the consumption of vegetables among WIC mothers and children.	Positive
			Milk	Consumption	Consumption of low-fat dairy products increased among Hispanic mothers and children and African American children.	
			Juice	Consumption	Consumption reduced in Hispanic mothers but no significant change in Hispanics or African American children or African American mothers.	
Andreyeva and Luedicke [17]	2015	New England States	F&V	Redemption	Increase in purchases of fresh fruits and both fresh and frozen vegetables from 2009 to 2010 among WIC households. Three times more spent purchasing fresh fruits than fresh vegetables.	Positive
Meiqari et al. [18]	2015	Atlanta	Milk	Consumption	Consumption of low-fat milk among WIC children significantly increased one to four weeks after the implementation but no significant change in WIC mothers’ consumption.	Positive
Morshed et al. [19]	2015	New Mexico	F&V	Consumption	Consumption of vegetables, excluding potatoes, decreased from 2008 to 2010 among preschool children in WIC households, while fruit consumption remained unchanged.	Positive
			Milk	Consumption	Consumption of lower-fat milk significantly increased.	
			Juice	Consumption	No significant change in the consumption of fruit juice.	
Reat et al. [20]	2015	Texas	F&V	Consumption	No observed change in fruit and vegetable consumption among WIC toddlers (1–2 y) between 2009 and 2011.	Positive
			Juice	Consumption	No significant change in juice consumption among WIC infants (6–12 m ^f^) and toddlers (1–2 y).	
			Infant F&V	Consumption	No significant change observed in the consumption of infant fruits and vegetables among WIC infants (6–12 m).	
Andreyeva et al. [21]	2016	New England States	Milk	Redemption	Decreased volume of whole milk purchased from 2009 to 2010 among WIC households.	Positive
			Juice	Redemption	Decreased volume of 100% juice purchased.	
Ishdorj and Capps [22]	2017	Texas	Milk	Consumption	Significant decrease in the consumption of whole milk among WIC children from 2008–2009 to 2010–2011, with an increase in the amount of lower-fat milk consumed.	Positive
			Juice	Consumption	The frequency of consumption of 100% juice increased.	
Zhang et al. [23]	2017	Virginia	Infant F&V	Redemption	Minority participants redeemed higher-priced brands of infant fruits and vegetables.	Positive
Guthrie et al. [24]	2018	National	F&V	Consumption	Among 12–23.9 m olds, fewer WIC children consumed fruit compared to both lower- and higher-income nonparticipants. No difference in the consumption of vegetables (higher-income 12–47.9 m).	Positive
			Milk	Consumption	Compared to non-WIC children, WIC children aged 12–23.9 m drank more whole milk, while children aged 24–47.9 m drank more low-fat and non-fat milk.	
			Juice	Consumption	WIC infants and children were more likely to consume 100% juice compared to non-WIC.	
			Infant F&V	Consumption	WIC 6- to 11.9-mo-olds were more likely to consume infant vegetables than lower-income nonparticipants. No difference was observed in fruit consumption.	
			Cheese	Consumption	No significant difference was observed in cheese consumption among WIC children and lower-income nonparticipants.	
Ng et al. [25]	2018	National	F&V	Redemption	Increased purchases of fruits and vegetables from 2008 to 2014 among WIC households with preschoolers.	Positive
			Milk	Redemption	Decreased purchases of high-fat milk.	
			Juice	Redemption	Decreased purchases of 100% juice.	
Vercammen et al. [26]	2018	National	F&V	Consumption	No difference in consumption of whole fruit and total vegetables between WIC children (2–4 y) and non-WIC children.	Positive
			Juice	Consumption	WIC children consumed significantly more 100% fruit juice than nonparticipants, exceeding the age-specific American Academy of Pediatrics maximum intake for juice.	
Charvet et al. [27]	2019	Florida	Milk	Consumption	No significant difference in the consumption of milk between non-Hispanic black and Hispanic WIC children (3–4.9 y).	Positive
			Juice	Consumption	WIC children consumed over twice the recommended amount of 100% fruit juice daily. Non-Hispanic Black children consumed more 100% fruit juice than Hispanic children.	
Hamner et al. [28]	2019	National	F&V	Consumption	WIC children (12–23 m) consumed more fruits and vegetables, excluding white potatoes, than non-WIC children.	Positive
			Juice	Consumption	WIC children consumed more 100% fruit juice than non-WIC children.	
			Milk	Consumption	No significant differences in consumption of whole, reduced-fat, low-fat, nonfat, or total milk by WIC participation status.	
Guthrie et al. [29]	2020	National	F&V	Consumption	WIC children (12–47.9 m) consumed more fruits and vegetables in 2016 compared to 2008.	Positive
			Milk	Consumption	A greater percentage of WIC children (12–47.9 m) consumed whole milk from 2008 to 2016, and a smaller percentage of WIC children (12–47.9 m) consumed reduced-fat milk. However, a greater percentage of WIC children (24–47.9 m) consumed more low-fat milk compared from 2008 to 2016.	
			Juice	Consumption	A smaller percentage of WIC children (12–23.9 m) consumed 100% fruit juice from 2008 to 2016. No significant change in the 100% fruit juice consumption in WIC infants and children (24–47.9 m).	
			Infant F&V	Consumption	WIC infants consumed more infant fruits and vegetables in 2016 compared to 2008.	
			Cheese	Consumption	A greater percentage of WIC children consumed cheese in 2016 than in 2008, with no statistical significance.	
Zhang et al. [30]	2022	Virginia	F&V	Redemption	Fresh fruits and vegetables accounted for 77.3% of CVB ^d^ redemptions. Non-Hispanic white and Black WIC households redeemed a smaller share of CVB than Hispanic households.	Positive
Fryar et al. [31]	2023	National	F&V	Consumption	The percentage of WIC children (1–4 y) who consumed whole fruit significantly increased from 2005–2006 to 2017–2018. No significant change in vegetable consumption.	Positive
			Milk	Consumption	WIC children had a significant increase in low-fat and non-fat milk consumption. But a lower percentage of WIC children consumed whole milk from 2005–2006 to 2017–2018.	

^a^ WIC = women, infants, and children. ^b^ F&V = fruits and vegetables. ^c^ SNAP = Supplemental Nutrition Assistance Program. ^d^ CVB = cash-value benefits. ^e^ y = year(s). ^f^ m = month(s). * Note: the quality of the included studies was assessed using the Academy of Nutrition and Dietetics guidelines for systematic reviews [8].

## Appendix A

The search strategy has been published on https://figshare.com/s/701320ae6604dd88ff3e (accessed on 25 February 2025).
